# Drug-Biopolymer Dispersions: Morphology- and Temperature- Dependent (Anti)Plasticizer Effect of the Drug and Component-Specific Johari–Goldstein Relaxations

**DOI:** 10.3390/ijms23052456

**Published:** 2022-02-23

**Authors:** Sofia Valenti, Luis Javier del Valle, Michela Romanini, Meritxell Mitjana, Jordi Puiggalí, Josep Lluís Tamarit, Roberto Macovez

**Affiliations:** 1 Grup de Caracterització de Materials, Departament de Física, Universitat Politècnica de Catalunya, EEBE, Av. Eduard Maristany 10-14, E-08019 Barcelona, Catalonia, Spain; sofia.valenti@upc.edu (S.V.); michela.romanini@upc.edu (M.R.); meritxellmitjana@gmail.com (M.M.); josep.lluis.tamarit@upc.edu (J.L.T.); 2 Synthetic Polymers: Structure and Properties. Biodegradable Polymers, Departament de Enginyeria Química, Universitat Politècnica de Catalunya, EEBE, Av. Eduard Maristany 10-14, E-08019 Barcelona, Catalonia, Spain; luis.javier.del.valle@upc.edu (L.J.d.V.); jordi.puiggali@upc.edu (J.P.); 3 Barcelona Research Center in Multiscale Science and Engineering, Universitat Politècnica de Catalunya, Campus Diagonal-Besòs, Av. Eduard Maristany 10-14, E-08019 Barcelona, Catalonia, Spain; 4 Institute for Bioengineering of Catalonia (IBEC), Baldiri Reixac 10-12, E-08028 Barcelona, Catalonia, Spain

**Keywords:** amorphous pharmaceuticals, polymer enantiomerism, Valium metabolite, formulation morphology, glass transition, dielectric spectroscopy, molecular mobility, secondary relaxations

## Abstract

Amorphous molecule-macromolecule mixtures are ubiquitous in polymer technology and are one of the most studied routes for the development of amorphous drug formulations. For these applications it is crucial to understand how the preparation method affects the properties of the mixtures. Here, we employ differential scanning calorimetry and broadband dielectric spectroscopy to investigate dispersions of a small-molecule drug (the Nordazepam anxiolytic) in biodegradable polylactide, both in the form of solvent-cast films and electrospun microfibres. We show that the dispersion of the same small-molecule compound can have opposite (plasticizing or antiplasticizing) effects on the segmental mobility of a biopolymer depending on preparation method, temperature, and polymer enantiomerism. We compare two different chiral forms of the polymer, namely, the enantiomeric pure, semicrystalline L-polymer (PLLA), and a random, fully amorphous copolymer containing both L and D monomers (PDLLA), both of which have lower glass transition temperature (*T*_g_) than the drug. While the drug has a weak antiplasticizing effect on the films, consistent with its higher *T*_g_, we find that it actually acts as a plasticizer for the PLLA microfibres, reducing their *T*_g_ by as much as 14 K at 30%-weight drug loading, namely, to a value that is lower than the *T*_g_ of fully amorphous films. The structural relaxation time of the samples similarly depends on chemical composition and morphology. Most mixtures displayed a single structural relaxation, as expected for homogeneous samples. In the PLLA microfibres, the presence of crystalline domains increases the structural relaxation time of the amorphous fraction, while the presence of the drug lowers the structural relaxation time of the (partially stretched) chains in the microfibres, increasing chain mobility well above that of the fully amorphous polymer matrix. Even fully amorphous homogeneous mixtures exhibit two distinct Johari–Goldstein relaxation processes, one for each chemical component. Our findings have important implications for the interpretation of the Johari–Goldstein process as well as for the physical stability and mechanical properties of microfibres with small-molecule additives.

## 1. Introduction

Mixtures of macromolecules with small organic molecules are ubiquitous in polymer technology. Many industrial polymer processes require the use of small-molecule solvents and plasticizers to tune the mechanical or thermal properties of polymers and thus their processability. The cosmetic, hygiene, paint, and food industries often rely on molecular solutions thickened by macromolecular additives. In the pharmaceutical industry, amorphous solid dispersions of small-molecule drugs in biocompatible polymers are studied for controlled drug release and for the design of drug delivery systems. In all these applications, it is crucial to determine and understand how the admixture of a small-molecule component to an amorphous or semicrystalline polymer affects the properties of the resulting product, in terms of processability, final morphology, and physical stability. Crucial aspects in this sense are the crystalline fraction and glass transition temperature of the mixture, and changes in macromolecular mobility and mechanical properties induced by the presence of the small-molecule additive.

The strive to replace synthetic polymers with more sustainable natural ones, and the growing concern with plastic pollution, have spurred extensive research on biopolymers derived entirely from renewable natural sources and biodegradable in the human body and/or in the environment [1]. Many biodegradable polymers, e.g., polylactide or poly(ε-caprolactone), have relatively low glass transition temperature (*T*_g_) and melting point compared to non-biodegradable ones. The low *T*_g_ of biodegradable polymers means that binary samples can be easily obtained where the small-molecule drugs with higher *T*_g_. Because the *T*_g_ of a binary mixture is generally intermediate between those of the two components [2,3,4,5], the drug could then act as a “genuine” antiplasticizing agent [6] for the macromolecule (as opposed to a purely mechanical antiplasticizer [7,8]), resulting in a higher *T*_g_ of the dispersion compared to the pure polymer. While understanding the fundamental properties of dispersions of poorly-water soluble drugs in biopolymers is a crucial prerequisite for their implementation as viable medications, to the best of our knowledge no systematic study has appeared on the glass transition temperature and molecular mobility of biodegradable polymer dispersions of drugs with slightly higher *T*_g_ than the macromolecular matrix.

Among biodegradable polymers, polylactide has high tensile strength, which makes it a versatile polymer for, e.g., drug delivery systems [9,10,11,12,13,14,15]. This polymer is constituted by repeating units that exist as two distinct (L- and D-) enantiomers, and the properties of polylactide samples depend on the distribution of such chiral units in the chains. In particular, the homopolymer poly-L-lactide (PLLA) consists mainly of L-lactide units and is semicrystalline, while the racemic poly-(D,L)-lactide (PDLLA) is totally amorphous due to the intrinsic disorder introduced by the uneven chirality of the repeat units.

In our contribution we employ dielectric spectroscopy to characterize different pure polylactide samples of different enantiomeric content and morphology, as well as polylactide dispersions of Nordazepam, a small-molecule drug which exhibits a slightly higher *T*_g_ than the biopolymer. We choose Nordazepam because it is a benzodiazepine derivative belonging to a large family of active pharmaceutical ingredients, of which the methylated form of Nordazepam, Diazepam, is the best-known example. Nordazepam, besides being a commercial drug, is also the main metabolite of Diazepam and an active metabolite of several other benzodiazepine drugs [16] in the human body. Contrary to Diazepam, it possesses a secondary amine group, which allows it to act as a H-bond donor and increases self-interactions, which in turn enhance the *T*_g_ of the demethylated derivative [17] (the *T*_g_ of Diazepam is lower than that of polylactide).

We choose a system with a low difference in glass transition temperature between the pure components, to avoid the difficulties inherent to binary mixtures with large *T*_g_ differences, which in general lead to structural and dynamic heterogeneity [18], and analyse distinct dispersions obtained either by modifying the enantiomeric content of the polymer or the preparation method (namely, solvent casting or electrospinning). Performing such a comparative study is necessary because distinct methods of preparation can lead to different physical properties of chemically equivalent solid dispersions [19,20,21].

We actually find that the sign of the (anti)plasticizing effect of the drug on *T*_g_ depends on the preparation method: while Nordazepam has a weak antiplasticizing effect on (fully amorphous) polymer films, it acts instead as a plasticizer for electrospun PLLA microfibres, reducing their *T*_g_ by as much as 14 K at 30%-weight drug loading. The resulting *T*_g_ is significantly lower than that of amorphous pure PLLA films, while electrospun fibres were semicrystalline, so that the plasticizing effect cannot be a consequence of a lower crystalline fraction. In fact, the effect of partial crystallinity on the fibres was opposite in sign and lead to an only modest increase in *T*_g_ of at most 1 or 2 K. We also characterize the kinetic fragility of the mixtures [22], which is a measure of how sensitive the structural relaxation time is to temperature changes in the vicinity of *T*_g_ [23] and influences the mechanical stability of a glass [24], and find a similar behaviour as that of *T*_g_. Finally, we investigate secondary relaxations, which are the only relaxation processes active at room temperature and deemed responsible for crystallization processes below *T*_g_ [25,26], to determine if the kinetic stability of the glassy samples may be increased compared to that of the pure polymer. We find that even homogeneous mixtures, characterized by a single *T*_g_ and a single structural relaxation above *T*_g_, display two distinct Johari–Goldstein relaxations, one for each chemical component.

## 2. Results

### 2.1. Characterization of Pure Polymer Samples

**Figure 1:**
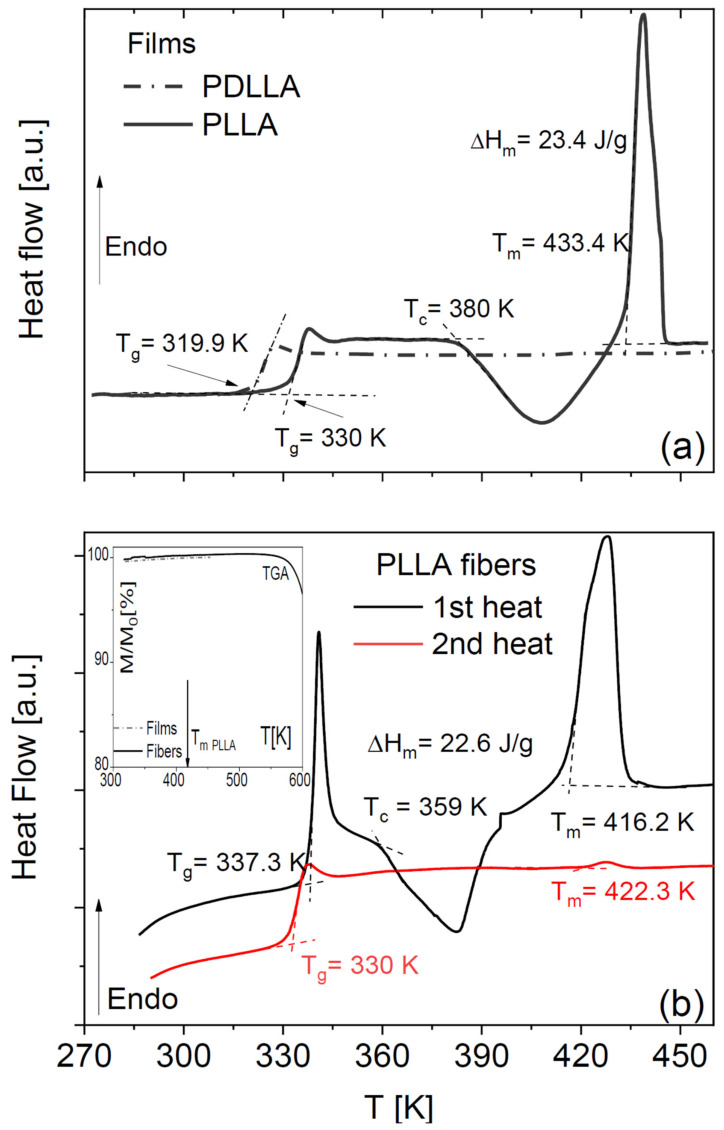
DSC thermograms, measured on heating, of PLLA and PDLLA films obtained by cooling from the melt (**a**), and of a scaffold of as deposited and annealed PLLA fibres (**b**). Dashed lines indicate the determinations of *T*_g_, *T*_c_ and *T*_m_. Inset to (**b**): TGA curves of pure PLLA films and fibres.

Pure amorphous PLLA films obtained by annealing a solvent-cast sample display the characteristic step-like increase in specific heat across its glass transition and then undergo (partial) cold crystallization, followed by melting at higher temperature (Figure 1a). Such peaks were absent in our home-synthesized racemic PDLLA polymer (same panel), which shows that it is completely amorphous as expected. It can be observed that the *T*_g_ of the fully amorphous PDLLA films is lower by 10 K with respect to that of the amorphous PLLA films obtained right after cooling from the melt. This difference in glass transition temperature is due to the different molecular weights of the samples (see Section 4).

The DSC thermograms of as-deposited scaffolds of PLLA microfibres (Figure 1b) have qualitatively the same line shape as the PLLA films but differ in the onset melting temperature (*T*_m_ = 433 K for films and *T*_m_ = 416 K for the fibre scaffolds) and in the onset temperature of crystallization, which were ca. 380 and 360 K for the films and the microfibres, respectively. Comparison of the enthalpy of cold crystallization and of melting with the melting enthalpy of fully crystalline PLLA (see Equation (3) in Section 4) allows concluding that the as-deposited fibres have a crystalline fraction of at most 2%, and the annealed ones a crystalline fraction of 21%, which is comparable with that of the cold-crystallized PLLA films.

The absence of any residual volatile solvents in either the membranes or the microfibres was checked by thermogravimetry analysis (see the inset to Figure 1b for representative traces for both morphologies), and by IR characterization (not shown).

Visual comparison of the DSC thermograms of the fibre scaffolds shows a significant ageing effect in the as-deposited fibres, visible both in the higher glass transition temperature (*T*_g_) and in the pronounced enthalpy recovery peak accompanying the first-heat scan. The higher *T*_g_ value could also be partially due to the non-zero crystalline fraction of the as-deposited fibres, since an increase in *T*_g_ with increasing crystalline fraction is often observed in semicrystalline polymers [27]. On the other hand, the *T*_g_ values of both PLLA fibres and films right after cooling them from above the melting point were identical (330 K). This shows that, to compare the *T*_g_ values of different samples, care must be taken to achieve a similar degree of ageing. In the following, to compare different sample morphologies, we will consider dielectric measurements carried out on samples with a similar thermal history.

**Figure 2:**
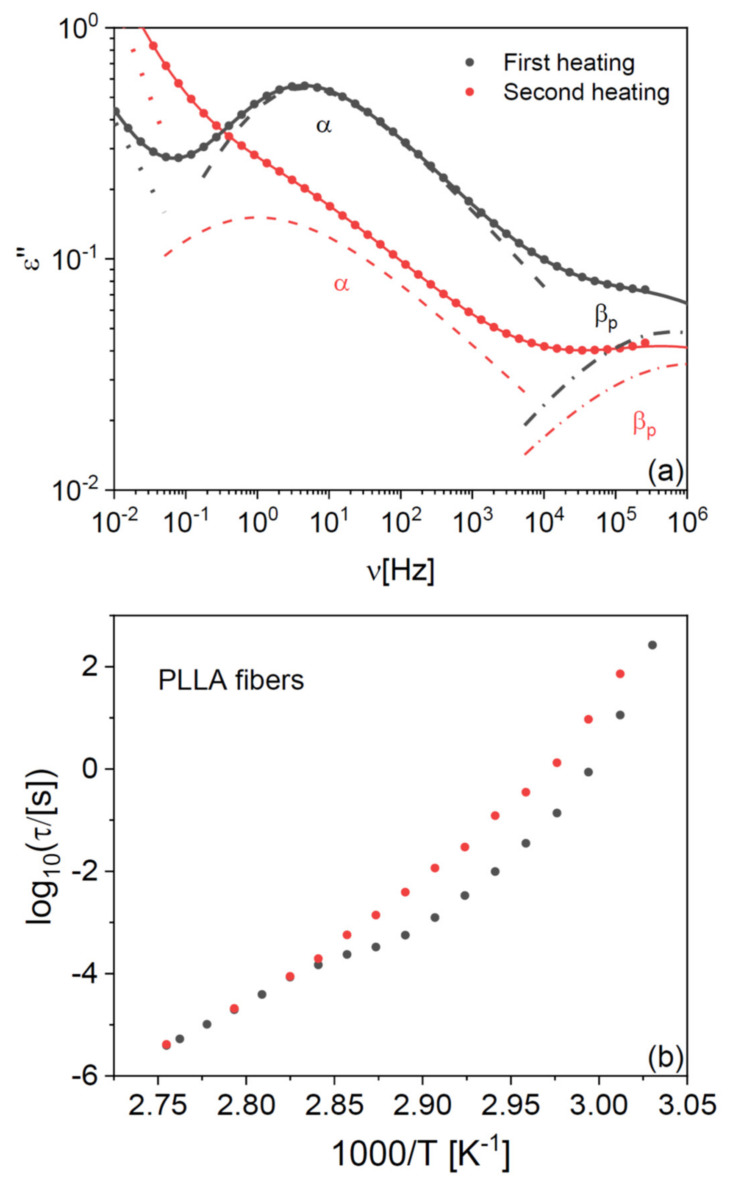
(**a**) Comparison of two isothermal dielectric loss spectra acquired at 338 K on the fully amorphous, as-deposited PLLA fibres (spectrum labelled as “first heating”, in grey) and on partially crystalline fibres obtained by annealing the sample to 373 K, above the cold crystallization temperature but below the melting point (“second heating”, in red). Markers are experimental points, and the solid line is the overall fit. Contributions to fits are also shown: the solid line represents the overall fit, the dotted line the conductivity contribution, the dashed and dash-dot lines the structural α relaxation and the secondary β relaxation of the polymer, respectively (see the text for details). (**b**) Arrhenius plot of the structural relaxation time of a scaffold of pure PLLA fibres, measured in two consecutive series of isothermal dielectric spectra acquired with increasing temperature.

In both PLLA films and fibres, the cold crystallization and melting peaks were almost completely absent in the second-heating thermograms, indicating that PLLA has only weak tendency to crystallize after melting also in microfibre form. To further investigate the impact of the crystalline fraction on the glass transition, we studied the effect of partial (cold) crystallization on the dielectric segmental mobility (structural α relaxation) of the PLLA fibres. For this purpose, two series of isothermal dielectric loss spectra were acquired on the scaffold of pure PLLA fibres while increasing the temperature in a step-like fashion, first on the as-deposited scaffold, and then on the same sample heated up to 373 K (i.e., above the cold crystallization temperature observed in Figure 1b) and cooled down again to below *T*_g_. Representative spectra at the temperature of 338 K, just above the *T*_g_ of the pure fibres, are shown in Figure 2a together with their fit with the imaginary part of Equation (4) (Section 4). The most intense spectral feature is the α relaxation, which is accompanied by a secondary relaxation at higher frequency (discussed in Section 2.4). The intensity of the α loss feature is clearly lower in the partially crystallized fibres, as expected from the reduction in the amorphous fraction. At the same time, the relaxation frequency (peak position of the loss feature) is lower, as expected due to the enhanced chain rigidity imposed by the presence of crystallites in semicrystalline polymers [28].

This effect is more clearly visible in Figure 2b, which displays the Arrhenius plot (logarithm of the relaxation time vs. inverse absolute temperature) of the structural relaxation time τ_α_ (see Equation (5) in Section 4), as determined from the fits of both series of isothermal spectra. The relaxation time was higher by almost a decade after partial crystallization compared to the as-deposited fibre, as visible by the vertical separation of the experimental points in Figure 2b.

The dielectric characterization allows studying the effect of crystallization on the structural relaxation in real time. In fact, the temperature dependence of τ_α_ for the as-deposited scaffold exhibited a cross-over in correspondence with the cold crystallization process, which was instead absent in the second temperature ramp on the same sample. Such cross-over in the relaxation time provides direct spectroscopic evidence of the slow-down of the cooperative α relaxation of PLLA upon partial crystallization of the fibre. The same effect has been observed in PLLA films [29], and as mentioned it is due to the enhanced rigidity of the amorphous fraction in the proximity to crystalline domains, as compared to the fully amorphous polymer [28].

Partial crystallization of the fibres also affects the dynamic glass transition temperature, defined by convention as the temperature at which the structural relaxation time reaches 100 s (i.e., log_10_(τ_α_/[s]) = 2). As will be shown in detail below, a fit procedure shows that the *T*_g_ of the partially crystallized microfibres is only 1 K higher than that of as-deposited ones. The small impact of partial crystallization on the *T*_g_ of PLLA fibres is consistent with previous results on PLLA films [29].

### 2.2. Morphological and Calorimetric Characterization of Binary Samples

**Figure 3:**
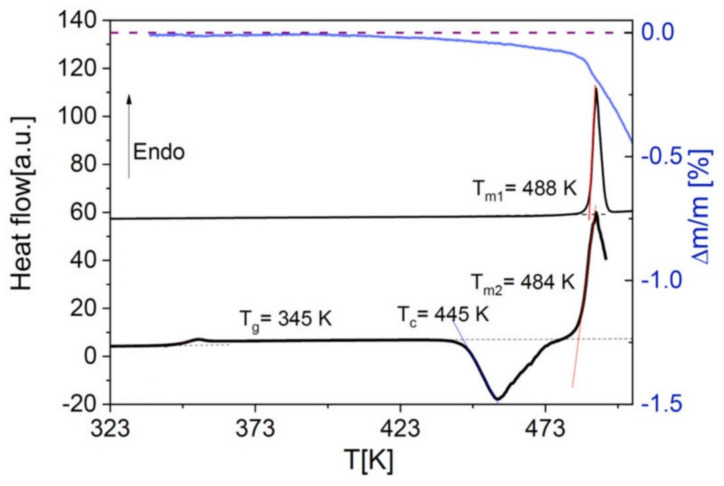
First and second DSC traces (left axis) and TGA trace (right axis) on heating of pure Nordazepam. The first DSC trace and the TGA trace were measured by heating the as-received crystalline powder. Mass loss of the drug starts already below the melting point *T*_m_. The second DSC trace was acquired after cooling from above *T*_m_ to below the glass transition temperature of the compound. Notice that *T*_m_ was lower in the second DSC trace (484 K) than in the first heating run (488 K), likely a consequence of the impurities produced by the partial degradation.

As mentioned in Section 4, the solvent-cast PLLA films were obtained by annealing at 435 K, i.e., above the melting point of the pure polymer. This was necessary both to remove all the solvent, and to ensure a fully amorphous and homogeneous sample, by cooling the PLLA matrix (or the PLLA-drug mixture) from the molten state [4]. The lack of any left-over solvents in annealed PLLA films was confirmed by thermogravimetry analysis and IR spectroscopy (not shown). Such annealing step is, however, unsuitable to achieve polymer dispersions of organic molecules that are thermally labile [30] such as Nordazepam, which decomposes prior to melting as visible in the thermogravimetry curve of Figure 3.

For this reason, we employed two different strategies (see Section 5 for details on sample preparation), namely: (*i*) casting fully amorphous films of the racemic PDLLA polymer, which only required heating to 393 K for the full evaporation of the solvent (instead of heating above the melting point of semicrystalline PLLA) [4]; (*ii*) electrospinning, directly from a co-dissolution, NOR-doped PLLA microfibres [31] with a small-enough diameter to ensure solvent evaporation during the formation of the fibres themselves.

**Figure 4:**
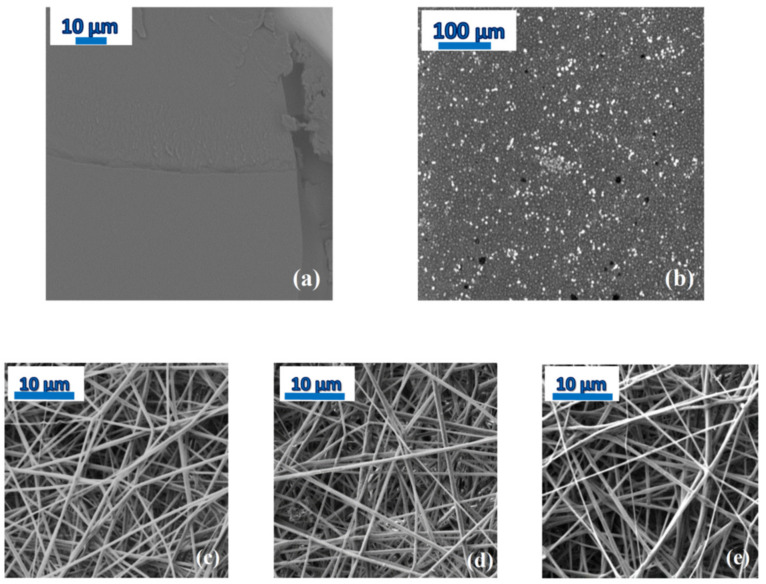
(**a**,**b**) SEM images of PDLLA films loaded with 20 wt-% (**a**) and 80 wt-% (**b**) of drug. While the 20 wt-% film appears completely homogeneous, the 80 wt-% film exhibits phase separation into polymer-rich and drug-rich domains. (**c**–**e**) SEM micrographs of pure PLLA fibres (**c**) and PLLA fibres loaded with 10 wt-% (**d**) and 30 wt-% (**e**) of Nordazepam. The scale of the image is indicated in the upper left corner of each panel.

Concerning strategy (*i*), because the racemic PDLLA polymer does not exhibit a crystalline form, heating was employed solely to extract the solvent from the bulk matrix [4]. The SEM micrograph of Figure 4a shows that the film of PDLLA with 20 wt-% NOR is homogeneous, while phase separation is visible in a PDLLA dispersion consisting of 80% NOR in weight (Figure 4b).

**Figure 5:**
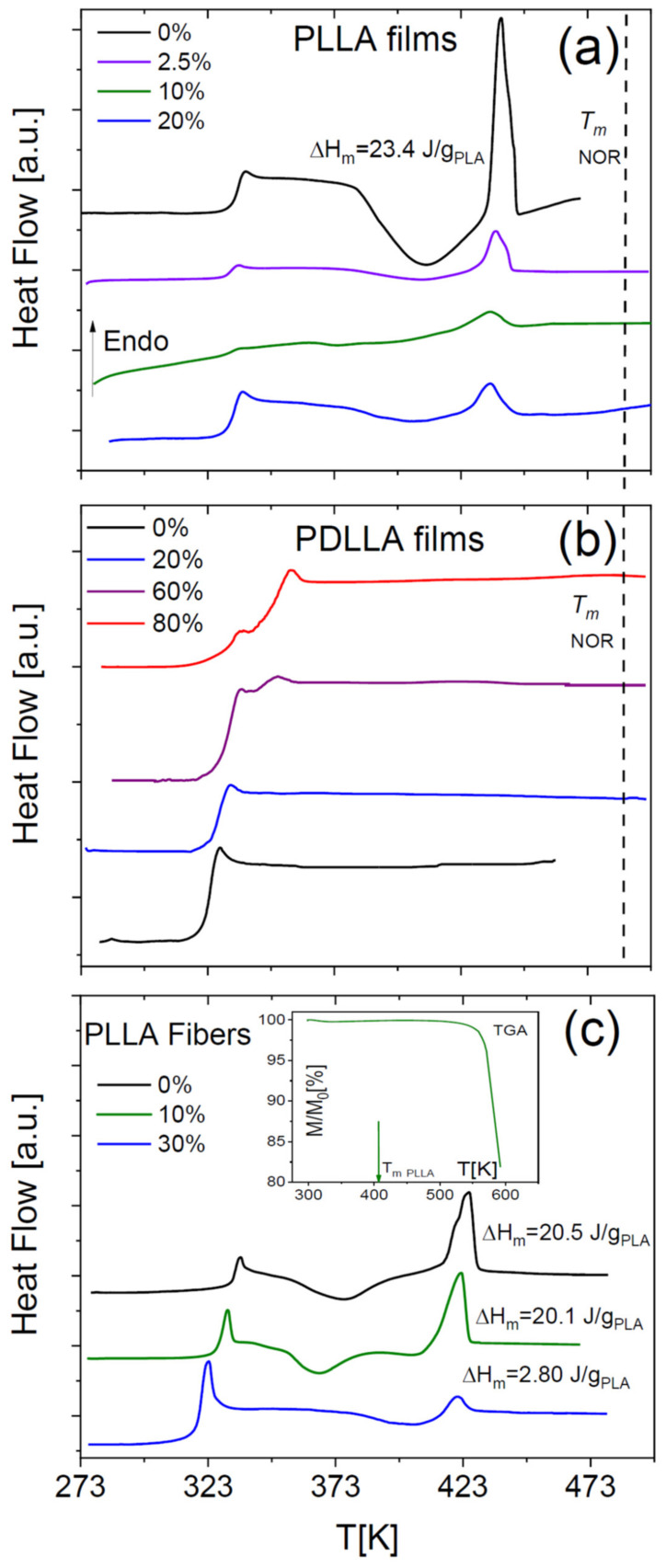
DSC traces on heating of films of NOR in PLLA (**a**) and PDLLA (**b**), and of electrospun microfibres of NOR dispersed in PLLA (**c**). The vertical dashed lines indicate the melting point of crystalline NOR, and the legends indicate the weight percentage of the drug in the samples. Indicated melting enthalpies refer to the mass of PLA (in grams) present in each sample. Inset to (**c**): TGA trace of the microfibre sample with 10 wt-% NOR loading.

On the other hand, in strategy (*ii*) the relatively small diameter of the microfibres (of the order of 600–700 nm, see the bottom panels of Figure 4) allowed full evaporation of the solvent during the electrospinning process and the subsequent storage under low vacuum conditions at room temperature (see Section 4), without any need for a subsequent annealing step. The lack of solvents is confirmed by the TGA trace of the scaffold of as-deposited microfibres, where no significant mass loss was detected below the onset of decomposition of the fibres (see the inset to Figure 1b for the pure PLLA fibres, and inset to Figure 5c for the fibres loaded with 10 wt-% NOR). Moreover, as it can be observed in the SEM micrographs of the as-spun microfibres (bottom panels of Figure 4), the fibres appear rather homogeneous, so that macroscopic phase separation can be excluded.

Figure 5a shows the DSC thermograms of NOR-doped PLLA films, acquired after supercooling the samples from 453 K down to their glass state. This temperature was chosen to be slightly higher than the melting onset of pure PLLA in order to ensure that the sample did not contain any PLLA spherulites or lamellae prior to supercooling. As visible in Figure 5a, the films exhibited a melting endotherm at a temperature similar to that of pure PLLA, preceded by an exothermic process signalling phase separation of the mixtures by crystallization of the polymer component. On the other hand, at higher temperature no melting peak ascribable to the pure drug could be observed (even at the relatively high drug concentration of 40%), which indicates that NOR is fully dissolved in the molten polymer matrix at these temperatures. In other words, even samples that are phase-separated at room temperature actually are homogeneous (viscoelastic) liquids at higher *T*. This validates our procedure for obtaining homogeneous dispersions in polylactide.

The DSC traces of binary films of NOR in racemic PDLLA are displayed in Figure 5b. These films exhibited a single *T*_g_ value at a low enough drug concentration (at least up to 20%), indicative of the formation of homogeneous amorphous solid dispersions (see also Figure 4a). On the contrary, films at higher drug loading were characterized by amorphous phase separation, with the occurrence of two distinct glass transitions (compare with the SEM micrograph in Figure 4b).

Our DSC and SEM characterization shows that both enantiomeric forms of the polymer lead to amorphous, homogeneous binary dispersions at low enough drug concentration. This result is analogous to that obtained with another small-molecule drug (the chloramphenicol antibiotic) dispersed in the same two polylactide polymers [4].

The DSC traces of the electrospun NOR-PLLA microfibres are shown in Figure 5c. A single *T*_g_ feature was observed at least up to a drug weight fraction of 30%, followed by a cold crystallization exotherm and corresponding melting endotherm, indicating partial crystallization of the PLLA fraction in the microfibres upon heating. In the case of 30% drug weight fraction, the melting enthalpy was considerably smaller than that of films or that of the microfibre with 10% drug weight fraction. This indicates that the crystalline fraction of the 30% fibre, even after cold crystallization, was significantly lower (<2%) than that of pure semicrystalline PLLA (21%) and shows that the crystallization of PLA was clearly hindered by the presence of drug molecules. No melting transition of the drug component was observed in any NOR-loaded PDLLA sample, indicating again that at high temperature NOR is dissolved in the molten polymer matrix. These results indicate that the drug and polylactide are highly miscible, at least at high temperatures.

**Figure 6:**
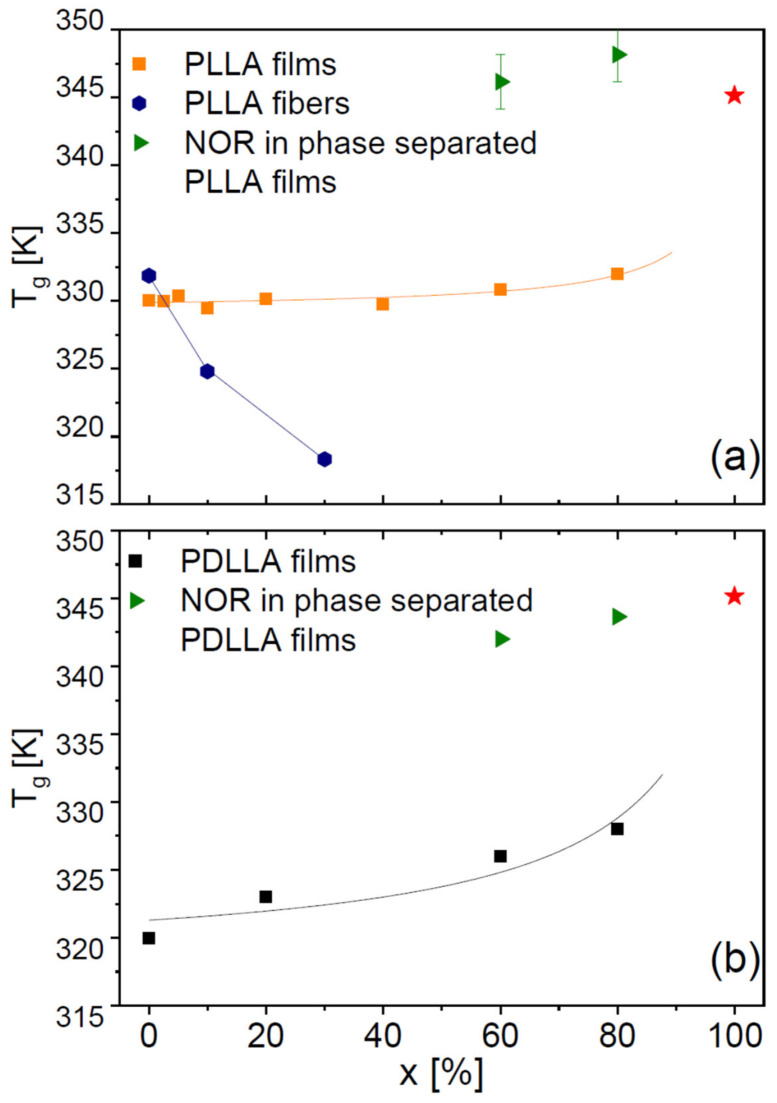
(**a**) Glass transition temperatures *T*_g_ of solvent-cast films (orange squares) and electrospun microfibres (blue circles) of PLLA, as a function of the mass content of drug (*x*). (**b**) *T*_g_ of NOR-loaded PDLLA films. In both panels, green triangles represent *T*_g_ values of the amorphous drug-rich regions of the phase-separated samples, and the red star corresponds to the pure drug. Lines are guides to the eye.

Figure 6 displays the onset glass transition temperature of all dispersions obtained with PLLA (both films and microfibres, panel a) and with PDLLA (panel b). In the case of the PLLA films, NOR has a weak antiplasticizing effect, the *T*_g_ of the binary films being slightly higher than that of the pure polymer (Figure 6a). The antiplasticizing effect of the drug is more pronounced in the PDLLA films (b). This is qualitatively in agreement with the expected behaviour of a binary mixture based, e.g., on the Gordon–Taylor equation [2], because the *T*_g_ of our home-synthesized PDLLA polymer is lower and thus further from that of NOR compared with PLLA: the greater the difference with the *T*_g_ of the organic molecule, the more visible the antiplasticizing effect for a given loading. In the PDLLA films with high NOR loading, which display amorphous phase-separation characterized by two distinct *T*_g_ values, the *T*_g_ of the component richer in Nordazepam is lower than that of the pure drug, again expected if the polymer acts, due to its lower *T*_g_, as an antiplasticizer for the drug.

On the contrary, in the PLLA microfibres (Figure 6a, blue circles) *T*_g_ is observed to *decrease* with increasing drug content, as can be gathered directly also from visual inspection of Figure 5c. This cannot be an effect of the solvent of the electrospun dissolution, not only due to the systematic dependence on drug concentration, but also because no mass loss is observed in TGA experiments (see the insets to Figure 1b and Figure 5c), indicating that no solvent is present (as also confirmed by IR, not shown). Similarly, it cannot be due to the crystalline fraction of the as-deposited microfibres, because the presence of a crystalline fraction increases the *T*_g_ compared to the fully amorphous polymer (by 1 K in the case of PLLA fibres, see Section 2.3) [28], while the observed *T*_g_ of the microfibre dispersions is actually lower than that of fully amorphous PLLA samples. In fact, as visible in Figure 6a, the *T*_g_ of PLLA microfibres with 30% drug loading is lower by 14 K with respect to the pure fibres, and lower by more than 10 K with respect to that of the fully amorphous PLLA films (both with and without drug) obtained by cooling from above the melting point of the polymer.

Thus, we find that Nordazepam actually has a genuine plasticizing effect on PLLA microfibres, while it has an antiplasticizing effect on the polylactide films. Such dependence upon the morphology of the sample is striking, and might reflect the peculiar chain morphology in the microfibres, where the PLLA chains are at least partially oriented in a parallel fashion (in the direction of the fibre axis) rather than randomly coiled as in the films [31]. We have acquired IR spectra of all samples (not shown) and did not find any significant difference in vibrational frequency of the IR bands between films and fibres, which indicates that drug-polymer interactions are similar in both morphologies. We suggest therefore that the antiplasticizing effect of the drug on the microfibres (and thus the breakdown of the Gordon–Taylor prediction) could be due to the structural changes induced by the drug molecules, which disrupt the quasi-parallel ordering of the macromolecular chains in the fibre morphology.

Concerning the antiplasticizing effect of the drug on the films, we point out that increasing the polymer’s *T*_g_ by adding a genuine antiplasticizing agent with higher *T*_g_, may be an interesting strategy to metastabilize amorphous or semicrystalline polymer dispersions in which demixing occurs by cold crystallization of the polymer component. In fact, the antiplasticizer effect of the drug entails that the segmental mobility of the polymer (see Section 2.3) is slower in the presence of the drug, and therefore that the nucleation and growth rates [32] of lamellae/spherulites are similarly slower. If phase-separation of the dispersions occurs by cold crystallization of the polymer component, as for example is the case of the chloramphenicol antibiotic dispersed in polylactide [4] but also of our dispersions (Figure 5a), an antiplasticizing effect of the drug would thus increase the kinetic stability of the amorphous mixture not only thanks to the entropy of mixing, but also by slowing down both the fluctuations leading to nucleation and the growth rate (which for temperatures just above the glass transition temperature is correlated with the structural (α) relaxation frequency [32]). It should also be noted, moreover, that a polymer in the (supercooled) viscoelastic liquid state might offer the advantage of a higher solubility of the molecular drug, as seems to be the case for the binary Nordazepam-PLA system.

### 2.3. Dielectric Spectroscopy of Binary Samples: Structural Relaxation

**Figure 7:**
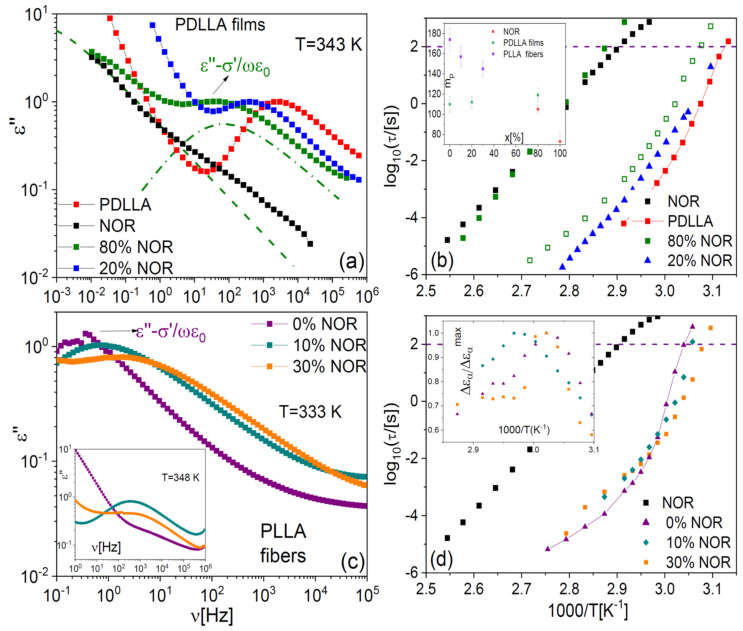
Isothermal loss spectra of PDLLA films at the fixed temperature of 343 K (**a**), and of as-deposited PLLA microfibres at 333 K (**c**), for different NOR loading. The inset to (**c**) displays the spectra of the same sample at 348 K. In (**a**), thin lines correspond to the fit components (primary α relaxations) of the spectrum of the phase-separated mixture with 80% weight drug content. (**b**,**d**) Arrhenius plot of the characteristic time of the α relaxation of the samples of panels (**a**,**c**). The lines connect the data of the pure polymer, to allow better visualization of the effect of drug loading. Inset to (**b**): kinetic fragility (Equation (2)) of the PDLLA films and PLLA fibres, as a function of the drug content. Inset to (**d**): dielectric strength of the primary relaxation in the microfibre samples, as a function of the inverse temperature.

To fully explore the impact of the molecular filler, we carried out temperature-dependent dielectric spectroscopy experiments on binary samples. At low drug content, all samples displayed a single structural (α) relaxation, as expected for a homogenous amorphous phase. Figure 7a displays the loss spectra of PDLLA films at different drug loading, at the same fixed temperature of 343 K. The vertical scale of each loss spectrum was normalized to the height of the dielectric loss feature that corresponds to the α relaxation, peaked in the kHz frequency range at this temperature. The structural relaxation is observed to shift to lower frequency with increasing drug content. Such a shift implies that the α relaxation of the PDLLA chains in the fibres slows down as a result of polymer-molecule interactions. The effect is consistent with the observed increase in the *T*_g_ of the films with increasing NOR content and confirms that the small-molecule drug acts as a genuine antiplasticizer for the polylactide films.

All dielectric loss spectra were fitted with model functions (see Section 4) to extract quantitative information on the characteristic time of relaxation processes. The Arrhenius plot of the structural relaxation time of polylactide films is displayed in Figure 7b. The genuine antiplasticizer effect of the drug on the polylactide films is again evident in the observed shift of the dynamic *T*_g_. It is worthwhile to note, however, that the antiplasticizing effect of the drug can be visualized more clearly by comparing the dielectric relaxation times in the Arrhenius plot, and that it is a consistent effect at all temperatures above *T*_g_. This is a clear advantage of dielectric spectroscopy compared to the DSC study of *T*_g_ values at a single fixed temperature-scanning rate. This advantage, together with the direct visualization of the impact of the drug on the structural dynamics offered by the loss spectra, shows the importance of performing dynamic studies to characterise the (anti)plasticizer effect of a polymer filler.

As visible in Figure 7a, the spectrum of the sample containing 80% in weight of NOR exhibited two main dielectric losses, of which the one at lower frequency matches the spectral position of the structural relaxation of pure NOR, also shown for comparison purposes. The latter spectrum was normalized so that the loss maximum of the α relaxation of pure NOR had the same intensity as the low-frequency feature of the 80% sample. The presence of two primary relaxations agrees with the observation of two distinct glass transitions in this phase-separated sample. The observed relaxation times indicate that one of the phases consisted of drug-loaded polymer domains, and the other of almost pure amorphous NOR. The loss feature of the sample with 20% drug had a dielectric strength (intensity) comparable to that of pure PDLLA, while the intensity of the lower-frequency feature in the sample with 80% drug was directly comparable to that of pure amorphous NOR, as it may be expected (not shown).

The spectra of the PLLA microfibres at the temperature of 333 K (Figure 7c) exhibited, compared with the films, an opposite shift of the segmental relaxation with increasing NOR content. The observed shift of the α relaxation toward higher frequency in the microfibre with NOR is the expected effect of a polymer plasticizer, in agreement with the DSC results presented in Section 2.2. It should be noted, however, that at higher temperature the α relaxation shifted to lower frequency with increasing NOR content, as visible in the isothermal spectra at 348 K displayed in the inset to Figure 7c. In other words, in the microfibres at high *T*, the drug displays again its “normal” antiplasticizer effect. Both effects are also visible in the Arrhenius plot of the structural relaxation time of the as-deposited fibres, shown in Figure 7d, where it may be observed that the sign of the shift changes from that of a plasticizer to that of a (genuine) antiplasticizer at the temperature of ca. 335 K.

If the plasticizing effect of the drug on PLLA microfibres is the result of the local structural modifications (disorder) induced in roughly parallel macromolecular chains, as we suggest, then the cross-over to the expected antiplasticizer effect may be rationalized considering that the activation of the segmental motion entails a dynamic disorder that makes irrelevant the (static) disorder induced by the drug molecules in the glassy state of the polymer.

As shown in the inset to Figure 7d, the dielectric strength of the samples displayed a maximum in correspondence with the cross-over temperature of 335 K. Different aspects concur to determine the dielectric strength of the cooperative relaxation, especially in polymers [5]; it is significant that the change in the temperature dependence of the dielectric intensity occurs precisely at the same temperature as the crossover between the opposite plasticizing behaviours.

**Figure 8:**
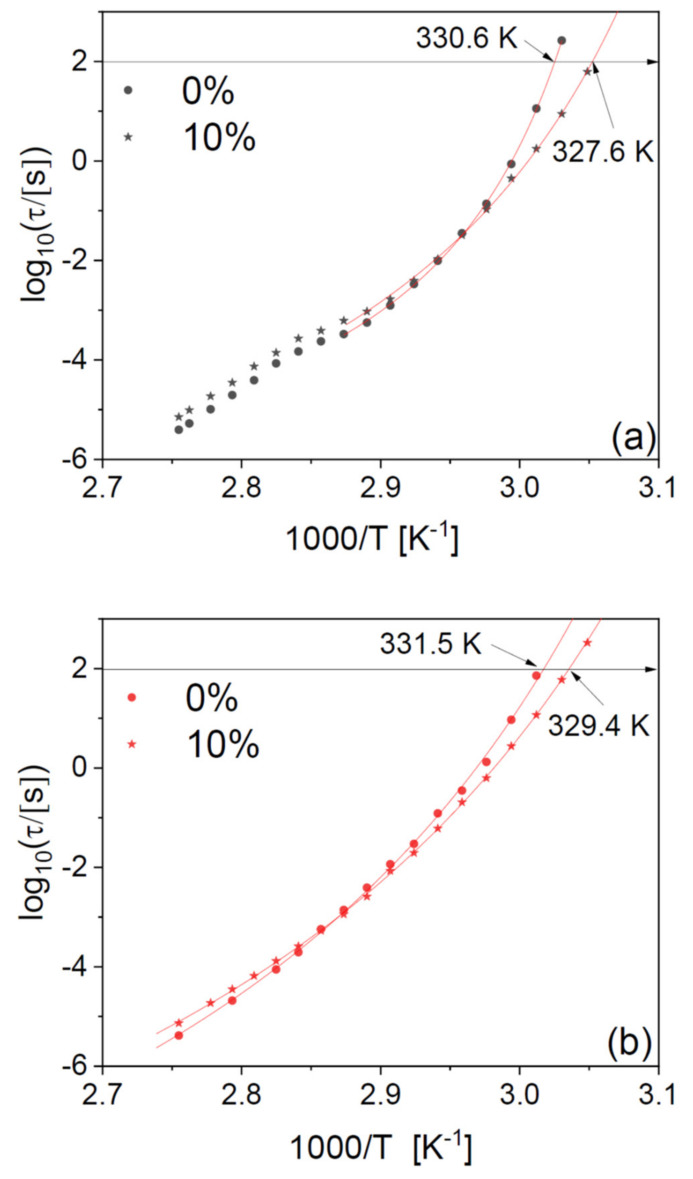
Arrhenius plot of the structural relaxation time τ_α_ of PLLA fibres loaded with 10-wt% NOR, compared with that of pure PLLA fibres, both for the as-deposited scaffolds (**a**) and after a first measurement ramp up to a temperature of 363 K (**b**) (i.e., above the onset of cold crystallization but well below the melting point). In both panels, markers are experimental points, continuous lines are fits with the Vogel–Fulcher–Tammann Equation (1), and the dashed horizontal line corresponds to τ_α_ = 100 s, which conventionally marks the dynamic *T*_g_.

It should be remembered that the PLLA microfibres undergo at least partial crystallization upon heating (see Figure 5c), and this is expected to have an impact on the relaxation time. Therefore, to investigate further the effect of the drug filler, we compare in panels (a) and (b) of Figure 8 the structural relaxation times of a PLLA microfibre scaffold at 10-wt% drug loading and those of a pure PLLA fibre, for two series of isothermal spectra acquired while increasing the temperature to 363 K, first on the as-deposited scaffolds (a), and then right afterwards, after cooling again to below *T*_g_ (b). The second series of spectra displays slightly higher dynamic *T*_g_ (by 1–2 K) with respect to the as-deposited scaffold, due to the effect of partial crystallization already discussed for the pure polymer fibres.

It can be observed that the plasticizing effect near *T*_g_ is present in both series of spectra, as is the cross-over between plasticizing and antiplasticizing behaviour at higher temperature. In particular, the plasticizer effect of the drug on the dynamic *T*_g_ is consistent (3 K) between the first and the second series of spectra, that is, it is maintained also after partial crystallization of the fibres.

In agreement with our findings on the pure fibres in Figure 2, the relaxation time of the drug-loaded fibres has a smooth temperature dependence in the second heat-up series (Figure 8b), while it clearly displays a discontinuity near the cold-crystallization temperature in the first one (Figure 8a). To discuss this discontinuity in more detail, we have fitted the Arrhenius plot of the observed relaxation times with continuous functions. The temperature dependence of the structural relaxation time in molecular and macromolecular glass formers is typically described by the Vogel–Fulcher–Tammann (VFT) function, which is given by [33,34,35]:

(1) $$\tau_{\alpha }\left(T\right)=\tau_{\infty }exp\left(D\frac{T_{VF}}{T-T_{VF}}\right)$$

Here, τ_∞_ is the segmental time extrapolated at a very high temperature, *D* is the so-called fragility strength coefficient, related with the kinetic fragility defined later on, and *T*_VF_ is the Vogel–Fulcher temperature. The VFT Equation (1) is mathematically equivalent to the Williams–Landell–Ferry equation. The temperature dependence of the relaxation time of the second series of spectra acquired on NOR-loaded microfibres (Figure 8b) could be well modelled with a single VFT function. In contrast, the as-deposited scaffold (Figure 8a) displayed two different temperature dependences above and below the cold crystallization temperature, due to the dynamic cross-over caused by the presence of crystalline domains (as already discussed in reference to Figure 2) [29].

It is interesting to analyse the so-called kinetic fragility index of the samples, which as mentioned in the introduction is a measure of how fast the relaxation time varies with temperature in the proximity of the glass transition. The kinetic fragility is defined as [23]:

(2) $$m={\left.\frac{d\left(\log_{10}\tau_{\alpha }\right)}{d\left(T_{g}/T\right)}\right|}_{T=T_{g}}$$

A high value of kinetic fragility implies that the molecular mobility changes fast when the temperature varies approaching *T*_g_; in general, this entails that a polymer dispersion with higher fragility is more sensitive to temperature fluctuations and therefore potentially more prone to crystallization. The fragility index, displayed in the inset to Figure 7b, is found to increase with the drug loading in the films (namely, from 110 to 119), despite the fact that the fragility of the Nordazepam glass former is actually lower than that of the amorphous polymer. On the contrary, the fragility decreases (from 174 to 145) with drug loading in the electrospun microfibres. In other words, the kinetic fragility exhibits the same behaviour with drug concentration as the glass transition temperature, and in particular, it has opposite dependence in the two studied sample morphologies. It is also interesting to note that the kinetic fragility of the pure PLLA microfibres is larger than that of the pure PLLA films, which may be due to the different chain morphology.

### 2.4. Johari–Goldstein Relaxations of Binary Samples

**Figure 9:**
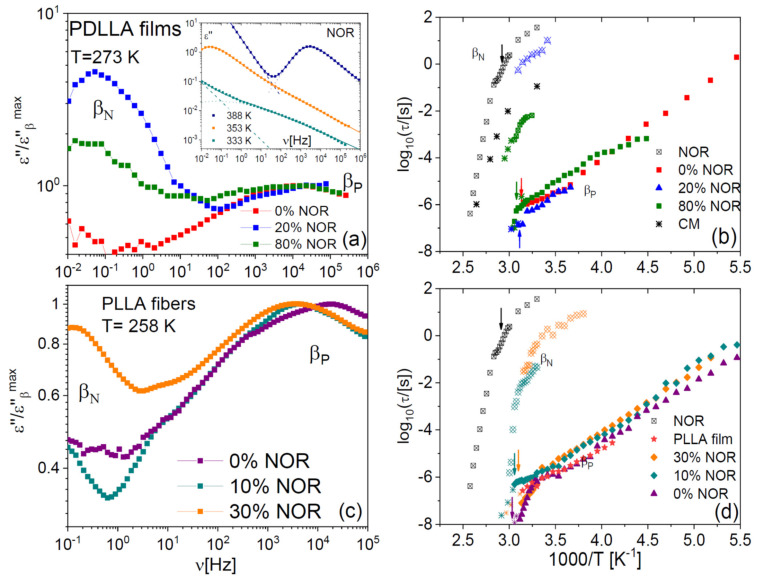
(**a**) Low-temperature dielectric loss spectra of PDLLA films with different NOR content, at the fixed temperature of 273 K. Inset to (**a**): isothermal loss spectra of pure supercooled liquid NOR, at selected temperatures. (**c**) Low-temperature spectra of PLLA microfibres with different NOR loadings, at the fixed temperature of 258 K. (**b**,**d**) Arrhenius plot of the secondary relaxation times τ_β_ of the films (**b**) and microfibres (**d**). Arrows indicate the glass transition temperature for each sample.

The studied binary samples also displayed secondary relaxations at lower temperature/higher frequency. The inset to Figure 9a displays representative low-temperature spectra of pure NOR. As we reported already in an earlier study [17], a secondary relaxation, labelled here as β_N_, is observed on the high-frequency flank of the α relaxation. Like the α process, the β_N_ relaxation shifted to higher frequency with increasing temperature. As visible in Figure 8a,c, a similar relaxation is observed also in the dispersions in polylactide, both in film (a) and microfibre (b) form. The Arrhenius plot of the β_N_ relaxation is displayed in panels (b) and (d) for both types of samples. The β_N_ process is significantly affected by the glass transition and by the composition of the dispersion. In particular, the β_N_ relaxation becomes faster with decreasing drug content, and its relaxation time displays a dynamic cross-over at the glass transition temperatures of the pure drug or of the mixture, respectively, which are indicated by vertical arrows in the right-hand side panels of Figure 8. In the case of the pure drug, the activation energy of the β_N_ relaxation changes from 3.5 eV above *T*_g_ to 0.8 eV below. Similar values are found in the binary samples.

As we discussed in a recent publication [17], in the pure drug, this relaxation corresponds to a non-diffusive relaxation of the whole molecule known as Johari–Goldstein (JG) β relaxation [36,37]. These secondary relaxations are observed in fully rigid molecules [38,39,40] and in polymers with monoatomic or methyl side groups such as polybutadiene and 1,4-polyisoprene [41,42] and correspond to rigid roto-translations of whole molecules or chain segments. The similar dependence of the β_N_ and α relaxation times with the chemical composition of the dispersions, and the crossover detected in the β_N_ relaxation times at *T*_g_, confirm the JG nature of this relaxation also in the dispersions. By contrast, local inter-conformer conversions are generally observed at a fixed frequency, regardless of the local environment [5]. In particular, the NOR molecule has two internal degrees of freedom, that correspond respectively to the ring-inversion dynamics of the diazepine moiety and the rotation of the benzyl ring around the σ-bond connecting it with the diazepine ring [17]. Both motions have a very weak dielectric intensity already in the pure compound, and they moreover occur at a much higher frequency than the β_N_ process [17]. These processes could not be detected in our samples due to the low drug content.

It is interesting to note that, in both the films and microfibres, the β_N_ relaxation times change in the same direction with increasing drug loading, namely, the β_N_ process becomes faster with decreasing drug content. This entails that the dispersion of the drug in the polymer matrix (and thus the dilution of inter-drug interactions) always lead to the same (plasticizing) effect on the non-diffusive JG relaxation of the drug, regardless of the sample morphology and thus of the impact of the drug on the segmental mobility.

The pure polylactide polymer also displays a secondary relaxation, which we label as β_P_ in Figure 9, and which a previous study has ascribed to local chain-twisting motions of angular amplitude of the order of 11º [43]. The β_P_ process is visible in roughly the same spectral range in all samples (both films and microfibres). It can be observed in Figure 9b,d that also this relaxation exhibits a dynamic cross-over near *T*_g_, as it is especially visible in the case of the microfibres. By comparing the relaxation times predicted by the Coupling Model [44,45] with the β_P_ relaxation times, we find that they are very similar, except in the pure polymer case. Both these features were encountered also in dispersions in PLLA and PDLLA of another drug (chloramphenicol) [4].

We thus confirm the conclusion of our previous studies [4,30], namely, that the β_P_ twisting mode of the polymer is the local, non-cooperative version of the segmental α relaxation of the polymer. This assignment is consistent with the idea that the JG relaxations correspond to small-angle single-particle rigid rotations taking place under strong spatial restrictions [46,47,48,49]. It thus appears that homogeneous “asymmetric” dispersions composed of relatively rigid macromolecular chains and small organic molecules and characterized by a single glass transition and primary relaxation, display not only one but actually *two* separate JG-like relaxations, one for each component.

## 3. Discussion

We explored two distinct enantiomeric forms of the polymer, PLLA and PDLLA, and two different fabrication methods from co-dissolutions, yielding two different sample morphologies, namely solvent-cast films and electrospun microfibres. The impact of the drug on the structural relaxation (macromolecular mobility) and thus on the dynamic and calorimetric *T*_g_’s of the dispersions is found to depend on the morphology: while Nordazepam has a genuine antiplasticizing effect on the polymer films, it acts as a plasticizer for the PLLA microfibres, reducing the *T*_g_ at 30% weight drug loading by more than 10 K with respect to the pure amorphous polymer. In comparison, the effect of the degree of crystallinity is much smaller, resulting in a modest increase in *T*_g_ (of the order of only 1 K) in semicrystalline fibres compared to amorphous ones.

The opposite plasticizer/antiplasticizer effect of the drug in the two morphologies is confirmed also by macromolecular mobility measurements, which further show that the sign of the plasticizing effect of Nordazepam on PLLA is temperature-dependent: while near *T*_g_ the segmental mobility (α) of PLLA is faster in the presence of the drug, as the temperature increases well above *T*_g_ the same dynamic process is slower in the presence of the drug than in its absence. Despite the fact that the homogeneous Nordazepam-polylactide binary samples exhibit only a single α relaxation, they all display two distinct Johari–Goldstein relaxation processes well-separated in frequency, one for each component. The Coupling Model predicts the Johari Goldstein relaxation time of the polymer component, which displays the same shift as the α relaxation time with increasing drug content. On the other hand, the polymer has a consistent plasticizing effect on the Johari Goldstein relaxation of the drug, independent of its effect on the structural relaxation.

None of the studied mixtures exhibited any signature of the melting transition of pure Nordazepam, even in samples that were initially phase-separated or that underwent demixing (cold crystallization) upon heating. The lack of any melting signature of the Nordazepam component indicates that the drug is homogenously dissolved in the polymer matrix when the latter is in the molten state (more precisely, above the melting point of the PLLA polymer, since the PDLLA polymer does not crystallize). This result suggests that mixing a drug with an amorphous biopolymer with relatively low *T*_g_ might prove beneficial for the stability of the resulting dispersion, since in the (liquid-like) viscoelastic state the equilibrium miscibility of the drug may be higher than in the glass state, as seems to be the case for the binary Nordazepam-Polylactide system.

Mixing a drug with a polymer with a lower glass transition temperature *T*_g_ could represent an interesting strategy to kinetically stabilize amorphous solid dispersions against crystallization. On one hand, the drug has a beneficial antiplasticizing effect in that it reduces the segmental mobility of the polymer and therefore its tendency to nucleate crystalline lamellae, which may prevent the occurrence of devitrification by cold crystallization of the polymer component. On the other, a polymer in the viscoelastic (liquid) state might offer the advantage of a higher solubility of the active pharmaceutical ingredient. The resulting mixtures are not strictly “solid” dispersions, but due to the viscoelastic properties of the polymer carrier they would be stiff enough for pharmaceutical applications.

## 4. Materials and Methods

Nordazepam (C_15_H_11_ClN_2_O, M_w_ = 270.71 g mol^−1^) was kindly provided by Bouchara-Recordati (France) with purity better than 99.5%. PLLA polylactide, a product of Natureworks (polymer 2002D), was kindly supplied by Nupik International (Polinyà, Catalonia, Spain). According to the manufacturer, this polymer has a D content of 4.25%, a residual monomer content of 0.3%, a molecular weight of approximately 180 kg mol^−1^, a glass transition temperature (*T*_g_) of 331 K and a melting point of 426 K. L-lactide with a purity higher than 98%, and D,L-lactide with a purity higher than 99%, were purchased by Sigma Aldrich and kept in a fridge at 277 K to avoid any degradation during storage. PDLLA was synthesized in our lab by melt-ring open polymerization of D, L-lactide in nitrogen atmosphere at 413 K for 24 h. Stannous octanoate was used as initiator of the reaction. The resulting polymer was dissolved in dichloromethane and precipitated in methanol. It was then filtered with a Buchner funnel and dried in a vacuum oven at ambient temperature for 48 h. Molecular weights (M_w_) and dispersity index (DI) were estimated by gel permeation chromatography (GPC) using a liquid chromatograph (Shimadzu, model LC-8A) equipped with an Empower computer program (Waters). A PL HFIP gel column (Polymer Lab) and a refractive index detector (Shimadzu RID-10A) were employed. The polymer was dissolved and eluted in 1,1,1,3,3,3-hexafluoroisopropanol at a flow rate of 1 mL min^−1^ (injected volume 100 μL, sample concentration 2.0 mg mL^−1^). The number and weight average molecular weights were calculated using poly(methyl methacrylate) standards. The synthesized PDLLA had M_w_ = 44.763 kg mol^−1^ and DI = 2.32. The results of the spectroscopic characterization (by NMR and IR) confirming the successful synthesis were published in an earlier study by some of us [4].

Binary mixtures of Nordazepam with polylactide were obtained from solution, due to the thermal liability of the drug, which decomposes upon melting (see Figure 3). PLA was dissolved in chloroform in a magnetic stirrer at 310 K for more than a day. Nordazepam was dissolved in dimethylformamide at a concentration of 10% *w*/*v*, and the correct amount of solution to achieve a desired drug loading was then added to the PLA/chloroform solution. Films were prepared by solvent casting directly on the desired supports, followed by heating to 393 K (PDLLA) or 435 K (PLLA), to ensure that all solvents evaporated and were thus absent in the dispersions, and in the PLLA case to melt the crystalline fraction formed during solvent evaporation. Various concentrations were prepared spanning from 2.5 to 80% *w*/*w* of NOR/PDLLA.

To prepare the microfibres, we first dissolved PLLA in a chloroform/acetone mixture (2:1 *v*/*v*) at a polymer concentration of 10% *w*/*v*. Acetone was employed as in our experience it enhances the formation of fibres via electrospinning. The desired amount of the Nordazepam solution in dimethylformamide was then added to the mixture to achieve fibres with weight percentage of drug of 10% and 30%. All electrospinning experiments were carried out at room temperature. Electrospun microfibres were collected on a target horizontal plane collector placed at different distances (10–25 cm) from the needle tip (0.84 mm inner diameter). We employed a high-voltage supply (Gamma High Voltage Research, ES30-5W) to apply to the collector a voltage between 10 and 30 kV. Polymer solutions were delivered using a KDS100 infusion syringe pump (KD Scientific, Holliston, MA, USA) allowing control of the mass-flow rate (from 0.5 to 10 mL h^−1^). Unloaded and Nordazepam-loaded microfibres were prepared using optimized parameters (height, voltage and flow). To ensure that all solvents are removed from the microfibres prior to their characterization, all microfibre scaffolds were allowed to dry under low vacuum conditions during at least 24 h at room temperature.

Scanning Electron Microscopy (SEM) images of both films and fibres were taken with a Focused Ion Beam Zeiss Neon 40 instrument, which is provided with a SEM GEMINI column with a Field Emission Schottky (4pA-20nA, 0.1–30 kV, resolution from 1.1 nm to 20 kV). Differential scanning calorimetry (DSC) and thermogravimetry (TGA) experiments were carried out in pierced aluminium pans under nitrogen atmosphere, by means, respectively, of a Q100 and a Q50 instruments from TA Instruments. Measurements were performed with a heating/cooling rate of 10 K/min and sample mass of around 10 mg. An upper limit to the crystalline fraction χ_c_ of as-deposited fibres was obtained from DSC measurements, namely, from the difference between the cold-crystallization enthalpy and melting enthalpy, normalized to the weight fraction (1 − *x*) of PLLA:

(3) $$\chi_{c}\left(\%\right)=100\;\cdot \;\frac{\Delta H_{m}-\Delta H_{c}}{\Delta H_{m}^{0}\left(1-x\right)}$$

Here, Δ*H_m_* and Δ*H_c_* are, respectively, the melting and the crystallization enthalpies of the as-deposited sample, and $\Delta H_{m}^{0}$ is the reference melting enthalpy (93.6 J g^−1^) for PLLA crystals having an infinite size [50].

A Novocontrol Alpha analyser was employed for broadband dielectric spectroscopy (BDS) measurements. The sample was placed in a stainless steel parallel-plate capacitor specially designed for the analysis of liquid samples, with the two electrodes kept at a fixed distance by means of silica spacers of 50 μm diameter. Temperature control of the sample capacitor was achieved with a nitrogen-gas flow cryostat (Novocontrol Quatro cryosystem) with an error not higher than 0.1 K. The solvent-cast films were heated to 393 (PDLLA) or 435 (PLLA) K prior to measurement, and were then rapidly cooled to 133 K. The fibre samples employed for dielectric characterization consisted of a bundle of microfibres pressed into a dense mat so as to achieve a denser scaffold with lower air content.

Isothermal spectra were acquired by increasing the temperature in a stepwise fashion, waiting each time 5 min for temperature stabilization. Spectra were recorded in the frequency range between 10^−2^ and 10^6^ Hz. To obtain relaxation times and quantify the changes in relaxation dynamics, we fitted the dielectric spectra (real and imaginary part simultaneously) as the sum of a power law representing the dc conductivity contribution and a Havriliak–Negami (HN) function for each relaxation component. The analytical expression of the HN function is [51]:

(4) $$\varepsilon^{*}\left(\omega \right)=\varepsilon_{\infty }+\frac{\Delta \varepsilon }{{\left(1+{\left(i\omega \tau_{0}\right)}^{a}\right)}^{b}}$$

Here ω = 2πν is the angular frequency, $\varepsilon_{\infty }$ is the permittivity in the high frequency limit, Δε is the dielectric intensity or strength, *a* and *b* are parameters describing the shape of the loss curves and τ_0_ is a time parameter connected to the characteristic relaxation time τ corresponding to the maximum of the relaxation time distribution. In terms of the fit parameters, τ is given by:

(5) $$\tau =\tau_{0}{\left[\sin\left(\frac{a\pi }{2b+2}\right)\right]}^{-1/a}{\left[\sin\left(\frac{ab\pi }{2b+2}\right)\right]}^{1/a}$$

The shape parameters *a* and *b* can vary between 0 and 1. A special case of the HN function is the Cole-Cole [52] function, which is obtained setting *b* = 1 in Equation (4), and for which τ_Cole-Cole_ = τ_0_. The primary structural relaxation of the samples displayed an asymmetric HN line shape and had a characteristic relaxation time τ_α_ given by Equation (5), while the secondary relaxations were broad and symmetric, and could be described with the Cole-Cole function.

## 5. Conclusions

We have presented a comprehensive study of a small-molecule drug (Nordazepam) dispersed in biodegradable polylactide, a binary system in which the polymer carrier has lower glass transition temperature (*T*_g_) than the pure drug. In particular, we have employed scanning calorimetry, broadband dielectric spectroscopy, and SEM microscopy to characterize Nordazepam dispersions in the form of both films and fibres. Calorimetry measurements are used to obtain *T*_g_ values as well as crystalline fractions of the samples, while dielectric measurements yield valuable information about (macro)molecular mobility, namely, on structural (α) as well as Johari–Goldstein (β) relaxation processes, and on the kinetic fragility of the α relaxation.

We study two enantiomeric forms of PLA: a homopolymer poly-L-lactide (PLLA) consisting mainly of L-lactide units, which is semicrystalline with calorimetric *T*_g_ = 330 K and a melting onset at *T*_m_ = 443 K; and a home-synthesized racemic poly-(D,L)-lactide (PDLLA), which is totally amorphous due to the intrinsic disorder introduced by the different chirality of the repeating units, and showed no melting point and a calorimetric *T*_g_ of 320 K due to its lower molecular weight compared to the PLLA sample. Drug-loaded fibres were obtained only with PLLA, while membranes were obtained with both polymers.

In both types of polymer membranes, but especially in the case of PDLLA films, the drug has a small but consistent antiplasticizer effect, visible in (*i*) an increase in *T*_g_ of a few K and (*ii*) an increase in the relaxation time of the structural α process (that is, a slowing-down of the (macro)molecular mobility). In marked contrast with this, the drug has an important plasticizer effect on the *T*_g_ of PLLA microfibres, resulting in a decrease in *T*_g_ of more than 10 K at 30% weight drug loading with respect to pure amorphous PLLA. Dielectric spectroscopy data confirm the increase in structural mobility (decrease in the structural relaxation time) near *T*_g_, and further show that the kinetic fragility index exhibits the same behaviour with drug concentration as does the *T*_g_, namely, it increases with the drug loading in the films, despite the fact that the fragility of the Nordazepam glass former is lower than that of the amorphous bulk polymer, while it decreases with drug loading in the microfibres.

Moreover, our dielectric results show that at higher temperature, when the structural relaxation is fully activated, the drug recovers its antiplasticizer behaviour (and the structural relaxation time of the loaded fibres is again higher than that of the pure fibres). This leads to suggest that the different sign of the plasticizer/antiplasticizer effect of the drug in the two different types of samples (membrane and microfibre) is associated with the different macromolecular chain morphology (more coil-like in the fully amorphous films, and more stretched in the partially crystalline microfibres).

Last but not least, comparison with the dielectric spectra of pure amorphous PLA and pure supercooled liquid Nordazepam samples shows the existence of two separate β Johari–Goldstein relaxations, one for each chemical component. This finding ha important implication for the identification of Johari–Goldstein relaxations as “precursor” processes for the structural relaxation.
